# Chemical characteristics and cancer risk assessment of smokeless tobacco used in Tunisia (neffa)

**DOI:** 10.11604/pamj.2021.40.45.24751

**Published:** 2021-09-17

**Authors:** Fatma Guezguez, Mohamed Abdelwaheb, Ichraf Anane, Saleheddine Rekik, Saad Saguem, Bassem Charfeddine, Sonia Rouatbi

**Affiliations:** 1 Department of Physiology and Functional Explorations, University of Sousse, University Hospital of Farhat Hached, Sousse, Tunisia,; 2 Heart Failure (LR12SP09) Research Laboratory, University of Sousse, University Hospital of Farhat Hached, Sousse, Tunisia,; 3 Applied Chemistry and Environment (UR13ES63) Research Unit, Department of Chemistry, Faculty of Sciences of Monastir, University of Monastir, Monastir, Tunisia,; 4 Department of Biophysics, Medicine Faculty of Sousse, University of Sousse, Sousse, Tunisia,; 5 Department of Biochemistry, Medicine Faculty of Sousse, University of Sousse, Sousse, Tunisia

**Keywords:** Smokeless tobacco, carcinogens, heavy metals, polycyclic aromatic hydrocarbons, risk assessment

## Abstract

**Introduction:**

neffa, a form of air-dried smokeless tobacco used in North Africa, is spuriously perceived as a lower risk alternative to smoking. The objective of this study was to provide information on some harmful constituents of neffa and to use them for cancer risk assessment.

**Methods:**

a high-performance liquid chromatography method coupled with fluorescence detector was used to determine polycyclic aromatic hydrocarbons (PAHs) in one sample of neffa. An atomic absorption spectrometry was performed to determine the concentrations of lead and cadmium in three samples of neffa. The levels of toxicants found in neffa were used to assess for lifetime cancer risk as advocated by the US Environment Protection Agency.

**Results:**

the determination of PAHs in neffa allowed the identification of phenanthrene and anthracene. However, the higher molecular weight PAHs such as Benzo(a)Pyrene B(a)P were not detected. The concentrations of cadmium and lead varied between 1.3 to 2.8µg/g and 1.7 to 4.6µg/g respectively. Cancer risk for cadmium and lead varied between 4.2E-03 to 9.3E-03 and 2.5E-06 to 6.4E-06 respectively. Cancer risk for Cd exceeded the range of 10E-04 to 10E-06 of an acceptable risk.

**Conclusion:**

neffa is not a healthy alternative for overcoming smoking addiction. It contains mineral and organic pulmonary toxicants. This study could serve as a scientific basis to inform consumers about the products´ toxicity and help them to quit smokeless tobacco (SLT) use.

## Introduction

Smokeless tobacco (SLT) use is a major health problem responsible for a large number of deaths globally. It affects more than 350 million people worldwide and the developing countries are bearing a substantial share of the burden [1]. SLT is highly addictive and damaging to health. The nicotine in this form of tobacco is more easily absorbed than by smoking, enhancing its addictiveness [2]. SLT comprises a wide variety of products with various local names [3]. In North Africa and especially in Tunisia, “neffa” is the main form of SLT. It consists in a homogeneous mixture made from Souffi tobacco leaves, a heavy and full-bodied tobacco, with food additives. Tobacco leaves are usually air-dried and finely chopped up. They are either inhaled throw the nose or held in the mouth between the gum and the cheek. In Tunisia, Neffa is manufactured but it could be produced artisanally by wildcrafters or farmers. Neffa consumption affects all age groups but it is more common among the elderly [4]. The prevalence of SLT use in 2016 was estimated at 5.4% and 2.3% among adults and adolescents respectively [5].

For several decades, studies on the negative health effects of tobacco focused on combustible cigarettes and so SLT health effects are less studied. This contributed to the spurious perception of SLT as having lower health effects. Smokeless tobacco contains many cancer-causing toxins and its use increases the risk of cancers of the head, neck, throat, oesophagus and oral cavity (including cancer of the mouth, tongue, lip and gums) as well as various dental diseases [1,2] and cardiovascular morbidity and mortality [1]. Indeed many potentially harmful constituents had been identified in SLT [6]. Cancers are related to polycyclic aromatic hydrocarbons (PAHs) and heavy metals. PAHs are formed during the thermal decomposition of organic molecules and their subsequent recombination. Yet, it has been reported that SLT may contain significant amounts of PAHs formed without any combustion [7] and a variety of toxic metals: arsenic, cadmium (Cd), lead (Pb) and nickel [6]. The identification of toxicants in SLT can provide accurate information for cancer risk assessment and can serve as a scientific basis to inform consumers about the products´ toxicity and help them to quit SLT use [8]. Given the diversity of products, production methods and the extent of production in the world, toxicant levels have great temporal and geographical variability. Thus, an accurate and complete characterization of the chemical composition of SLT is recommended for each country and region [9]. For the author´s best knowledge no previous study has assessed toxicological risk of neffa produced in Tunisia. The aim of the present study was to determine the chemical composition of the neffa in PAH and selected heavy metals and to use them for cancer risk assessment according to the USEPA principles.

## Methods

**Neffa sample collection:** seven samples of neffa were purchased in the region of Sousse. Collected neffa included two samples produced by the national tobacco and matches board and five artisanal products. All the samples were transferred into plastic bags and stored in a dry place prior to analysis. Exported neffa through illegal routes from neighbouring countries was excluded from this study. Finally one sample was selected for PAHs determination and three samples were selected for heavy metals determination.

**Determination of PAHs:** eleven standard PAHs (antracene, benzo(a)pyrene, benzo(b)-fluoranthene, benzo(k)fluoranthene, chrysene, dibenzo(a,c)antracene, dibenzo(a,h)antracene, fluoranthene, indeno(1,2,3)pyrene, phenanthrene and pyrene) were used. The determination of the mentioned PAHs was performed on a 10g sample of neffa mixed with 20mL of hexane. A centrifugation and purification of the mixture was carried out and the resulting solution was concentrated to 1mL by vaporization. A high-performance liquid chromatography (HPLC) (HP 1050, Hewlett Packard, Waldbronn, Germany) method coupled with fluorescence detector was performed as previously described by Belkhiria *et al*. [10]. The chromatographic separation of PAHs was performed using a C18 reversed phase column (Agilent; Hypersil ODS, 5 mm, 4 mm 250 mm, Santa Clara, CA, USA). The mobile phase used was acetonitrile-water at a flow rate of 1mL/min at 30°C. Fluorescence detection was carried at λ excitation (250 nm) and λ emission (395 nm). The chromatographic separation of PAHs was performed after a direct injection of 20 μL of the prepared sample.

**Determination of heavy metals:** from each sample, 0.5 g was used for the digestion process. Each weighed sample was placed in a digestion vessel and mixed with 6 mL of 60% nitric acid, 2 mL of 97% sulphuric acid and 6 mL of 35% hydrogen peroxide. The mixture was heated on a sand bath for 60 min and then allowed to cool for 15 min. Thereafter, the digested samples were diluted with 25 mL of nitric acid in volumetric flasks. It was then filtered with Whatman filter paper [11]. Concentrations of Cd and Pb in the digested sample solutions were determined by an atomic absorption spectrometer (HR-CS AAS - Analytic Jena AG).

**Assessment of potential toxicity:** the toxicant concentration was compared to the Gothiatek® standards, where the maximum allowable levels and limits of certain harmful constituents (PAHs, heavy metals...) in Swedish SLT products have been set [12]. The toxicant daily intake (TDI) expressed in μg/g was calculated using the following equation:

*TDI=Ctoxicant x NDI*

*TDI* is the toxicant daily intake, *Ctoxicant* is the concentration of toxicant (μg/g) and *NDI* is the estimated neffa daily intake (g/day) (assuming 10 intakes a day, 1 g per intake). The estimated *TDI* for each sample was expressed in absolute value (μg/day) and as a percentage of permissible daily intake according to the joint Food and Agriculture Organization and World Health Organization (FAO/WHO) [13]. For the assessment of potential toxicity (life time cancer risk) of neffa, the US environment and protection agency (USEPA) principles were applied [14] and the following equation according to Ayo-Yusuf *et al*. was used [8]:

*Life time cancer risk = ADE lifetime x CPE*

*ADE lifetime* is the lifetime average daily exposure (mg/kg bodyweight/day), this was calculated as follows assuming an individual with a body weight of 60 kg, with 30 years of consumption out of 70 years lifespan:


$$ADE\text{ lifetime}=\frac{TDI\times 10^{-3}}{body\text{ weight}}\times \frac{number\text{ of years of consumption}}{\text{average lifetime}}$$

*CPF* is the cancer potency factor (mg/kg body weight/day)^-1^, this was retrieved from the University of California´s (Berkley) carcinogenic potency database. CPF of B(a)P, Cd and Pb are 1.1, 46.1 and 0.02 (mg/kg bodyweight/day)^-1^ respectively. The bioavailability of Cd and Pb in humans varies between 5 to 15% and 10 to 15% respectively and it depends on the root of administration. When exposure is by inhalation, the absorption of Cd and Pb could reach 50% and 100% respectively [15,16]. To be consistent with the literature available about cancer risk assessment for SLT, we assumed an absorption fraction in the systemic circulation, referred to as transfer of 6% [8]. The calculated cancer risk was considered high if it exceeded the standard of the US Environmental Protection Agency (USEPA) of an acceptable cancer risk in the range of 10^-4^ to 10^-6^.

## Results

**Figure 1:**
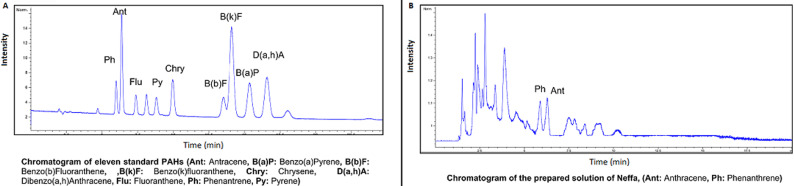
A) chromatographic separation with fluorescence detection of 11 standard polycyclic aromatic hydrocarbons; B) and that of the prepared solution from neffa

The chromatographic separations of 11 standards PAHs with fluorescence detection and that of the solution prepared from neffa are shown respectively in Figure 1. Two PAHs, phenanthrene and anthracene, were detected. The other carcinogenic PAHs were not detected. The Cd and Pb concentrations in the different neffa samples are shown in Table 1. All samples exceeded the maximum allowable levels of heavy metals in SLT products by more than two to six folds. The estimated daily intake of metals in the tested neffa is shown in Table 2. We notice that neffa provides near the half of the permissible daily intake of cadmium. Table 3 shows the calculated cancer risk related to neffa for Pb and Cd assuming a transfer of 100% and 6%. The calculated cancer risk attributable to Cd is greater than the “acceptable risk” range of 10^-6^ to 10^-4^.

**Table 1:** determination of heavy metals´ concentration in the different neffa samples compared to the gothiatek® standards

	C_Cd_ (μg/g)	C_Pb_ (μg/g)
**Samples**		
Sample 1	1.3	4.5
Sample 2	2.0	4.6
Sample 3	2.8	1.7
Gothiatek standards	0.5	1.0

C_Cd_: concentration of cadmium in μg per gram of neffa; C_Pb_: concentration of lead in μg per gram of neffa

**Table 2:** estimated daily intake of metals of the tested neffa compared to the permissible daily intake limits for the human consumption of the heavy metals according to the joint Food and Agriculture Organization and World Health Organization (FAO/WHO)

	Cd	Pb
Estimated daily intake of metals provided from neffa in μg/day and in percentage of the permissible daily intake (%)		
Sample 1	12.9 (21.5)	45.1 (15.0)
Sample 2	19.8 (33.0)	45.7 (15.2)
Sample 3	28.4 (47.4)	17.4 (5.8)
Permissible daily intake according to the FAO/WHO in μg/day	60	300

Cd: cadmium; Pb: lead

**Table 3:** estimated cancer risk related to neffa use

Neffa	Cd		Pb	
	100% transfer	6% transfer	100% transfer	6% transfer
Sample 1	4.25 x 10^-3^	2.55 x 10^-4^	0.64 x x10^-5^	0.38 x 10^-6^
Sample 2	6,52 x 10^-3^	3.91 x 10^-4^	0.65 x 10^-5^	0.39 x 10^-6^
Sample 3	9.36 x 10^-3^	5.61 x 10^-4^	0.25 x 10^-5^	0.15 x 10^-6^

Cd: cadmium; Pb: lead

## Discussion

The main findings of the current study were: 1) neffa is not a healthy alternative for overcoming smoking addiction. It contains inorganic (Pb and Cd) and organic (phenanthrene and anthracene) pulmonary toxicants; 2) the calculated cancer risk attributable to Cd is high and exceed the range of 10^-4^ to 10^-6^ of an acceptable risk.

The PAHs constituents usually result from combustion of organic molecules, whereas here neither manufacturing procedures nor consumption required combustion. It has been previously reported that leafy plants such as tobacco contain high levels of PAHs [7]. These constituents are absorbed from the soil. Their presence reflects contamination of the environment from natural sources and anthropogenic activities [7]. Nevertheless, the carcinogenic effect of phenanthrene and anthracene remains uncertain. According to the IARC, these substances are unclassifiable as to their carcinogenicity to humans [10]. Theses identified PAHs are lower molecular weight. The main health concerns arise from the larger high molecular weight PAHs such as B(a)P which is class 1 carcinogenic (IARC) [7]. They were not identified in this studied sample. This result is expected since manufacturing procedures are based on air-drying. Indeed, previous studies have found that the larger high molecular weight PAHs were much higher in the fire-cured SLT compared to that air-cured [17,18].

The current study also confirmed that neffa contained high levels of heavy metals. Their concentrations exceeded by several folds the maximum allowable levels set for SLT according to the Gothiatek® standards. The calculated cancer risk attributable to Cd is considerably higher than the 10^-6^ to 10^-4^ range of “acceptable risk” advocated by the USEPA. These data are parlous and corroborate those of the literature [8,11,19,20] (Table 4). Furthermore, the SLT composition in metal is subject to a great variability between regions and even for the same region, which might spurious toxicological assessment. These types of variations are related to differences in production procedures between countries. The unconscionable use of pesticides and fertilizers constitute the main contributor to agricultural soil and plants pollution. Tobacco plants are prone to accumulate metals absorbed from the soil in their leaves. The accumulated metals results from a complex interaction between the surrounding environment of plants, soil and animals [11].

**Table 4:** available data about metals´ levels in SLT and their estimated lifetime cancer risk (transfer 6%)

Study	Country	SLT	Cd concentration (μg/g)	Cd lifetime CR	Pb concentration (μg/g)	Pb life time CR
Ayo-Yusuf and Connolly 2011	India	Medicinal nicotine gum (nicorette)	NR	2.8^-3*^	NR	2.5^-6*^
Saeed et al. 2012	Pakistan	30 Naswar	2.34* [0.25 - 9.2]‡	[0.78 - 109.42]‡	21.05* [12.4 - 11.5]‡	[0.063 - 0.573]‡
Song et al. 2016	USA	7 moist snuf	1.28* ± 0.14†	2.17^-4*^ ± 2.29^-5†^	0.46* ± 0.19†	3.35^-8*^ ± 1.41 E-08†
Hossain et al. 2018	Bangladesh	5 Gul	[1.05 - 3.06]‡	[2.53-3 - 8.17^-3^]‡	[1.16 - 1.74]‡	[4.4^-8^ - 6.6^-8^]‡
The present study	Tunisia	3 Neffa	[1.29-2.84]‡	[2.55^-4^ - 5.61^-4^]‡	[1.73 - 4.5]‡	[0.15^-6^ - 39^-6^]‡

Cd: cadmium; lifetime CR: lifetime cancer risk; NR: not reported; Pb: lead; SLT: smokeless tobacco; data were expressed as *means, †standard deviations or ‡ranges

Health hazards arising from exposure to metals are extensively documented. Cadmium exposure may cause skeletal damage, kidney damage with tubular dysfunction or even renal failure. Cadmium is also a human carcinogen that has been associated with prostate and renal cell cancers as well as head and neck cancer [15]. Khlifi *et al*. reported that patients presenting with laryngeal and nasopharyngeal cancers had high blood cadmium levels compared to healthy subjects. The highest blood cadmium levels were found among neffa consumers and smokers [21]. Inhaled Cd through smoking or chewing tobacco, increases the risk of head and neck cancers and related disorders. Lead (Pb) is a cumulative toxicant with several deleterious systemic effects on cardiovascular, neurological, renal, skeletal, haematological and respiratory systems [16]. The route of lead (Pb) exposure is an important determinant of its toxicity. Indeed, absorption of ingested Lead is about 10-15% while inhaled lead is almost completely absorbed [16].

Smokeless tobacco contains many cancer-causing toxins and its use increases the risk of cancers of the head, neck, throat, esophagus and oral cavity as well as various dental diseases. Toxicological risk assessment principles were applied in this study. They estimate the cancer risk associated with exposure to carcinogens, in order to provide information for consumers about the products´ toxicity and to form a rational basis for regulatory actions to reduce specific toxicants in SLT products [7]. Risk assessment is an established four step process [22], the first step consists in hazard identification or identification of harmful compounds expected to be in tobacco, these compounds have been well identified by the US Food and Drug administration and presented as a list of harmful and potentially harmful constituents of tobacco [6]. In our study, only PAHs, lead and cadmium were screened for. It has been previously reported that the Tunisian SLT contained high levels of toxicants such as nickel, aluminium and chromium and found that local SLT was the most hazardous compared to the other forms of tobacco (conventional cigarettes and water pipe) [23]. Further studies to identify the tobacco specific N nitrosamine in neffa are steel needed for a more complete characterization of the products. The second step consists in exposure assessment which means quantification of the extent, frequency and duration of exposure to the population of interest. It is based on numerous assumptions responsible for a substantial share of the uncertainties associated with the estimate. Nevertheless, selecting the upper-bounds values of the exposure parameters (daily intake, duration of consumption, transfer…) ensures that the resultant risk is not underestimated [22]. Since data from human are lacking, data from investigations in animals are indispensable and can provide useful information about relative carcinogen potency [24]. However, there is a difference between the dose rates of the toxicant administered by oral route required for tumour induction in the rat and that required in human. Indeed, it has been found that the risk derived from rodent assays are at least one order of magnitude too high [24]. The final step consists in cancer risk characterization which is based on combination of toxicity and exposure data. This reports on the magnitude of uncertainty associated with the estimates. On the other hand, SLT contain numerous toxicants that occur together and their interaction cannot be rigorously predicted. The USEPA assumed that toxicants act additively and cancer risk should be evaluated by summing cancer risk deriving from each toxicant [22]. Other harmful constituents of SLT were not identified in this study, the calculated cancer risk arising from neffa use is probably underestimated.

The authors recognize that this work has some limitations: the small number of the studied neffa samples and the partial identification of mineral and organic toxicants in neffa. Yet, this study could serve as a scientific basis for more complete studies with larger sample size, representative of the Tunisian market.

## Conclusion

Neffa is not a healthy alternative for overcoming smoking addiction. It contains mineral and organic toxicants with deleterious health effects and an unacceptable risk of cancer. These results can provide information about toxicological risk of SLT often unknown by the consumers and may present grounds to implement a national control programme aimed to reduce neffa consumption. This study may serve as a scientific reference to enable policy-level discussion to reduce toxicants in the produced SLT which should be subject to the same regulation as cigarettes and other tobacco products.

### What is known about this topic

- Smokeless tobacco contains many cancer causing toxicants.

### What this study adds

- Neffa contains high levels of heavy metals with an unacceptable risk of cancer.
